# Transcriptional Program of Ciliated Epithelial Cells Reveals New Cilium and Centrosome Components and Links to Human Disease

**DOI:** 10.1371/journal.pone.0052166

**Published:** 2012-12-31

**Authors:** Ramona A. Hoh, Timothy R. Stowe, Erin Turk, Tim Stearns

**Affiliations:** 1 Department of Biology, Stanford University, Stanford, California, United States of America; 2 Department of Genetics, Stanford University School of Medicine, Stanford, California, United States of America; University of Pittsburgh, United States of America

## Abstract

Defects in the centrosome and cilium are associated with a set of human diseases having diverse phenotypes. To further characterize the components that define the function of these organelles we determined the transcriptional profile of multiciliated tracheal epithelial cells. Cultures of mouse tracheal epithelial cells undergoing differentiation *in vitro* were derived from mice expressing GFP from the ciliated-cell specific *FOXJ1* promoter (*FOXJ1:GFP*). The transcriptional profile of ciliating GFP+ cells from these cultures was defined at an early and a late time point during differentiation and was refined by subtraction of the profile of the non-ciliated GFP- cells. We identified 649 genes upregulated early, when most cells were forming basal bodies, and 73 genes genes upregulated late, when most cells were fully ciliated. Most, but not all, of known centrosome proteins are transcriptionally upregulated early, particularly *Plk4*, a master regulator of centriole formation. We found that three genes associated with human disease states, *Mdm1*, *Mlf1*, and *Dyx1c1*, are upregulated during ciliogenesis and localize to centrioles and cilia. This transcriptome for mammalian multiciliated epithelial cells identifies new candidate centrosome and cilia proteins, highlights similarities between components of motile and primary cilia, and identifies new links between cilia proteins and human disease.

## Introduction

Cilia are highly conserved microtubule-based structures that are found in all branches of the eukaryotic tree [1]. The ciliary axoneme is nucleated and anchored at the cell membrane by a modified centriole called the basal body, and is surrounded by a specialized membrane that is continuous with the plasma membrane. There are several types of cilia, differing in the specifics of their structure and function. The primary cilium, present in one copy on many cell types in the body, has a 9+0 axonemal structure and is important for sensing chemical and mechanical stimuli. Important developmental signaling pathways such as *hedgehog* and non-canonical *Wnt* are transduced through primary cilia [2], [3]. Motile cilia usually have an additional central pair of microtubules in the axoneme (9+2), as well as accessory structures including dynein arms and radial spokes associated with ciliary beating [4]. In addition to generating force, motile cilia also have sensory functions [5], [6], [7], [8].

Centrioles are a conserved feature of all organisms that have ciliated cells, and one of the main functions of centrioles is to serve as basal bodies [9], [10], [11], [12], [13]. In most cycling cells, centrioles lie within the larger centrosome, an assemblage of many proteins that functions as a microtubule organizer and a signaling center. Centrioles duplicate once per cell cycle and segregate at mitosis, maintaining the capacity for cilium formation in most cells. Although a number of human diseases have now been linked to defects in centriolar or centrosomal proteins, cell division can often proceed normally in the absence of centrioles, and adult flies generated without centrioles are viable but have defects associated with an inability to make cilia [9], [14]. We consider that it is most useful to consider the centrioles, centrosome and cilium as a single functional complex in animal cells, and will refer to it as the centrosome/cilium when appropriate.

Genetic defects in the centrosome/cilium are associated with a range of phenotypes in humans, consistent with their broad set of functions. The most clear-cut defects are those associated with ciliary motility. Motile cilia are found both in single copy, in sperm and in specialized epithelial cells in the embryonic node, and in hundreds of copies, on the surface of ciliated epithelial cells lining the respiratory airways, the ventricles of the brain, and the oviduct. Defects in motile cilia result in failure of embryonic turning, respiratory failure, infertility, hydrocephalus and randomized left-right asymmetry [15]. More recently it has been found that there is a set of human disease phenotypes that are caused by defects in the centrosome/cilium, but which are distinct from those strictly related to motile cilia [16]. These phenotypes include retinal degeneration, obesity, sterility, polydactyly, polycystic kidney disease and mental retardation. The diversity of pathologies reflect the important roles that cilia play in a multitude of tissues in the body [15], and in many cases the nature of the connection between centrosome/cilium and phenotype is not understood. Collectively, diseases caused by an underlying defect in cilia are termed ciliopathies.

Previously, we examined the process of basal body and cilium formation in multiciliated tracheal epithelial cells (MTECs) [17], [18]. Multiciliated cells are unique in that they produce 200–300 cilia and centrioles during differentiation, whereas most G1 phase cycling cells have two centrioles, and either lack a cilium or have a single primary cilium. Most of the centrioles in multiciliated cells are generated by a little-studied process in which multiple centrioles grow simultaneously from the surface of deuterosomes, structures of unknown origin [19], [20]. This duplication process is, at least outwardly, different from that in cycling cells, in which single new centrioles grow from the sides of the two existing centrioles [13]. Despite the differences in function and manner of formation, basal bodies and cycling cell centrioles share many of the same components [17]. Moreover, depletion of SAS-6 or CEP120, which are required for centriole formation in human and mouse cells [18], [21], blocked basal body generation in multiciliated cells [17], [18].

To further understand centrosome/cilium structure and function, we here exploit the multiciliated epithelial cell system by determining the transcriptional profile of ciliating cells during differentiation. This approach combines the *in vitro* culture model that we previously described [17] with a transgenic mouse that expresses GFP from the ciliated cell-specific promoter of the human forkhead-box transcription factor FOXJ1 [22]. The mouse tracheal epithelium is a pseudostratified epithelium that consists of a complex mixture of cells including basal cells, goblet cells and multiciliated cells. In culture, ciliogenesis is initiated by the establishment of an air-liquid interface (ALI) [23] and the process proceeds in discrete stages including the formation of basal body precursors in the cytoplasm, the migration of nascent basal bodies to the apical surface of the cells, docking at the plasma membrane and axoneme extension [17]. Importantly, ciliogenesis in culture is semi-synchronous, such that most FOXJ1-expressing cells at four days after ALI (ALI+4) are in the process of basal body formation, whereas by ALI+12, most are fully ciliated [17].

We find that the set of upregulated genes in multiciliated epithelial cells reveals the similarities between primary and motile cilia, and suggests how this cell type is uniquely able to generate hundreds of centrioles and cilia. In addition, we identify new components of these structures, including previously uncharacterized proteins and proteins associated with human disease. These results establish new links between the centrosome, cilium and genetic diseases with poorly understood molecular etiology.

## Materials and Methods

### Animals and Animal Care

MTECs were derived from wild-type C3H X C57Bl/6J F1 hybrid or *FOXJ1:GFP* transgenic mice (a gift from L. Ostrowski, University of North Carolina at Chapel Hill, Chapel Hill, NC), which were generated on C3H X C57B1/6J F1 hybrid background [22]. Heterozygous *FOXJ1:GFP* mice were obtained as described [17]. PCR genotyping was performed using a forward primer specific to the *FOXJ1* promoter region (5′-GCAGGCACCACATACTTATTCGGAGG-3′) and a reverse primer specific to GFP (5′-CGTCCTTGAAGAAGATGGTGCG-3′). Mice were sacrificed by CO_2_ anesthesia and tracheas were surgically removed. All work was approved by the Stanford University Administrative Panel for Laboratory Animal Care (SUAPLAC Protocol-11659) and was performed in accordance with SUAPLAC guidelines.

### MTEC Culture and Sorting

MTECs for the microarray experiments were derived from tracheas removed from male and female mice between 6–20 weeks of age from multiple litters. For microarray experiments, MTECs were pooled and cultured on 6-well plates containing Transwell-Clear permeable membrane supports (Corning) for the microarray experiments. Cells dissociated from the tracheas of approximately 12–15 mice were seeded per 6-well plate. Culture and differentiation of MTECs was performed as previously described [17]. For each biological replicate, fourteen to sixteen wells were pooled and sorted for ALI+4; four to six wells were pooled for ALI+12. Technical replicates were performed using RNA from the same sample on a separate array. To prepare MTECs for FACS, cells were first removed from the filter by incubating at 37°C for 30 minutes in a 1∶1 mixture of Cell Dissociation Solution (Sigma-Aldrich) and 0.5% Trypsin/EDTA (Invitrogen). After washing 2X in PBS, cells were resuspended in PBS at 10^6^ cells/ml and passed through a 100 µm nylon mesh cell strainer (BD Falcon) to remove clumped cells. GFP+ and GFP- (from ALI+12) populations were collected in serum. GFP+ MTECs derived from *FOXJ1:GFP* mice had at least 10-fold higher FITC intensity than the mean FITC value of MTECs derived from wild-type *FOXJ1* ^−/−^mice. Sorting was performed at the Stanford Shared FACS Facility on a Vantage Vanford sorter. Data were acquired using CellQuest software.

### RNA Extraction

Total RNA was extracted using the RNeasy Kit (Qiagen) according to manufacturer’s protocols. Briefly, FACS sorted samples in serum were diluted 5∶1 in PBS and centrifuged for 5 minutes at 1200×g. The pellet was resuspended in 250 µl of Buffer RT and spun through a QiaShredder Column (Qiagen) to disrupt cells before proceeding with the rest of the protocol. RNA was eluted in 30 µl of RNase free water. Total RNA concentration was measured by NanoDrop ND-1000 (NanoDrop Technologies, Wilmington, DE) and stored at –80°C until amplification.

### Probe Preparation

Amplification and coupling of RNA to Cy3/Cy5 was performed with amino allyl MessageAmp II aRNA amplification kit (Ambion) according to the manufacturer’s protocols. Briefly, total RNA from MTECs or Universal Mouse Reference RNA (Stratagene) were reverse transcribed using the supplied T7 oligo-dT primer to produce first-strand cDNA. After second strand synthesis, cDNA was subjected to a single round of in vitro amplification with amino allyl-modified UTP. The modified RNA was purified, dried and coupled to either Cy3 or Cy5 reactive dyes. Dye incorporation and final probe concentration was checked by NanoDrop-1000 and 2–10 µg labeled RNA was fractionated using RNA Fragmentation Reagent (Ambion) before proceeding to hybridization.

### Microarray Hybridization and Image Extraction

Hybridization was done on MEEBO oligonucleotide arrays printed at the Stanford Functional Genomics Facility (www.microarray.org). MEEBO slides were post-processed the same day of hybridization. Fluorescently labeled amplified RNA samples from MTECs were hybridized to the array with an amplified reference RNA sample (Stratagene) for 16 hours at 65°C. Arrays were scanned on an Agilent G2565AA scanner immediately after washing. Features were extracted using GenePix6.0 (Axon Instruments). Detailed microarray protocols are available at http://brownlab.stanford.edu/protocols.html .

### MIAME

Data were submitted to Gene Expression Omnibus (GEO) according to MIAME (Minimum Information About a Microarray Experiment) guidelines and can be accessed at GSE42500.

### Data Analysis

Linear Models for MicroArray (LIMMA) LIMMA software [24], an R-based program the employs Empirical Bayes for the analysis of microarray data, was used to identify differentially expressed genes and for data normalization. LIMMA software is available from the BioConductor website http://www.bioconductor.org/packages/release/bioc/html/limma.html. Genes were clustered by Pearson Correlation using Cluster 3.0 and visualized using TreeView as implemented by SMD.

Functional annotation of differentially expressed genes was performed using bioinformatics tools found online at DAVID (Database for Annotation, Visualization and Integrated Discovery, version 6.7, from the National Institute of Allergy and Infectious Diseases (NIAID), NIH (http://david.abcc.ncifcrf.gov/)) [25]. Genes that passed a fold-change cutoff of 2-fold or greater in addition to an adjusted *P* value <0.05 in the subtracted MTEC transcriptome from ALI+4 and ALI+12, or in the GFP- non-ciliated cells, were uploaded to DAVID, and functional groups that are overrepresented in these sets of genes relative to the human genome were identified.

### Plasmids

cDNA encoding TTC12 (Accession BC067297) was obtained from Open Biosystems. cDNA encoding MDM1 was PCR amplified from a cDNA library generated from MTEC cultures harvested at ALI+10. The MTEC cDNA library was generated using Superscript II RT (Invitrogen) according to the manufacturer’s protocol. cDNAs were cloned in frame to pEGFP-N1 (Invitrogen) to generate C-terminal GFP fusion expression constructs, or to the lentiviral transfer vector pRRL.sin-18.PPT.PGK.IRES.GFP.pre [17] using either the Age I site or the Age I site in combination with Bam HI. All PCR generated clones were sequenced to ensure in-frame fusion to GFP. The rat DYX1C1-GFP fusion in the pCAGGS plasmid was a gift from Dr. J.J. LoTurco. The cDNA library was generated using Superscript II RT (Invitrogen) according to the manufacturer’s protocols.

### Cell Culture and Transfection

Mouse embryonic fibroblast NIH/3T3 cells (ATCC Catalog CRL-1658) and hTERT-immortalized human retinal pigment epithelium hTERT-RPE1 (ATCC Catalog CRL-4000) cells were cultured in DMEM with 10% FBS (Invitrogen). For transfection, cells were plated onto poly-L-lysine coated coverslips at 80% confluency the night before transfection. The day of transfection, cells were rinsed 2× with PBS and incubated with media containing plasmid plus Lipofectamine 2000 (Invitrogen) for 6 h before replacing with fresh media. Cells were imaged 24–48 h later.

### Indirect Immunofluorescence

NIH/3T3 cells and MTECs were prepared for indirect IF by washing twice with PBS and fixing in 4% PFA at room temperature for 10 min. or methanol at −20°C for 10 min. After fixation, cells were rinsed with PBS and blocked in PBS-BT (PBS +3% BSA +0.1% Triton X-100) for 30 min. at room temperature. MTEC filters were cut at this point from the solid supports for staining. MTECs were incubated with primary antibody for 1 h at room temperature, while tissue culture cells were incubated for 30 min. at room temperature. Incubations with secondary antibodies were for 30 min. at room temperature for all cells. To visualize DNA, cells were incubated with 4′,6-diamidino-2-phenylindole (DAPI, Molecular Probes) for 5 min. at room temperature using a 1 µg/ml dilution for NIH/3T3 cells and a 10 µg/ml dilution for MTECs. In between incubations, cells were washed 3× with PBS-BT for 5 minutes each. The primary antibodies used were mouse anti-acetylated alpha-tubulin (1∶2000, Sigma), mouse anti-gamma-tubulin (1∶500, Sigma), mouse anti-MLF1 (1∶400, Abnova) and rabbit anti-polaris (1∶500, gift from Bradley Yoder). The secondary antibodies used were Alexa 488 and 594-conjugated goat anti-mouse and goat anti-rabbit (1∶200, Invitrogen). All antibodies were diluted in PBS-BT. Cells were then observed under a Zeiss Axiovert 200M microscope (Zeiss, Thornwood, NJ) using a 100× oil-immersion objective.

## Results

### Transcriptional Profile of Ciliogenesis in Tracheal Epithelial Cells

**Figure 1:**
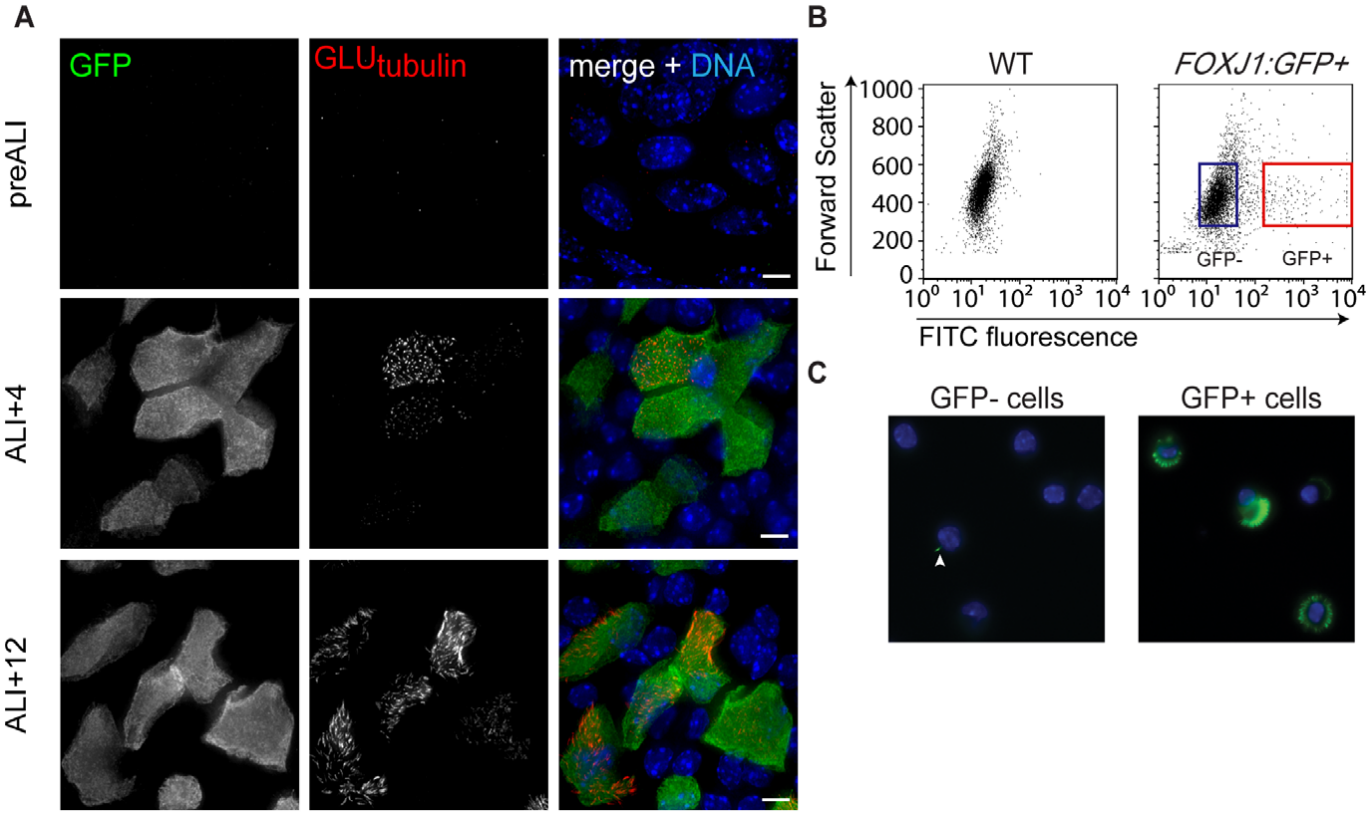
Microarray analysis of ciliating mouse tracheal epithelial cells. ( A) Immunofluorescence staining showing representative images of GFP expression and ciliogenesis in *FOXJ1:GFP* MTEC cultures before establishment of the air-liquid interface (pre-ALI), at 4 days after ALI (ALI+4) and at 12 days after ALI (ALI+12). MTEC cultures were stained with antibodies against GFP, against glutamylated tubulin to mark cilia and basal bodies, and with DAPI to mark nuclei. GFP is expressed from the *FOXJ1* promoter early during ciliogenesis, but is not expressed pre-ALI. At ALI+4, most *FOXJ1:GFP+* cells are undergoing centriole formation but have not formed cilia. At ALI+12, most *FOXJ1:GFP+* cells are forming cilia or have completed ciliogenesis. Scale bars, 5 µm. (B) FACS analysis of cells dissociated from a trachea from a wild type mouse (left panel) and a trachea from a *FOXJ1:GFP*-expressing mouse (right panel). The red and blue rectangles are representative gates used to sort GFP+ and GFP- populations, respectively. (C) Sorted cell populations were stained with DAPI (blue) and combined acetylated alpha-tubulin and gamma-tubulin antibodies (green) to detect cilia and basal bodies. After sorting, 90–95% of cells in the GFP+ population at ALI+12 had observable cilia and or amplified basal bodies, whereas <1% of cells in the GFP- population stained positive for these markers, although some had a single primary cilium (arrowhead).

We used microarrays to identify genes that are differentially expressed in mouse tracheal epithelial cells (MTECs) differentiated *in vitro*. The cells were derived from a mouse strain expressing GFP under control of the *FOXJ1* ciliated cell-specific promoter [22]. This promoter is activated early in the differentiation process [17] (Figure 1A), allowing the use of fluorescence activated cell sorting (FACS) to sort dissociated cells from the MTEC cultures into ciliating and non-ciliating populations (Figure 1B). MTECs were harvested at two time points after transition to an air-liquid interface culture (ALI), which induces differentiation of ciliated cells in the columnar epithelium [26]. The two time points chosen were based on previous analysis of landmark events in these cultures [17]; four days after ALI (ALI+4) to enrich for genes involved in initial steps of centriole duplication, and ALI+12 to enrich for genes expressed when cilia are mature (Figure 1A). At ALI+12 the majority of cells expressing GFP are fully ciliated, with a small fraction of cells at an earlier stage of ciliogenesis [17]. Importantly, the *in vitro*-differentiated epithelium accurately recapitulates the complexity of the *in vivo* tissue with respect to gene expression [27], with several cell types in addition to the ciliated epithelial cells of interest to this study. To separate ciliated from non-ciliated cells in our analysis, single-cell suspensions of MTECs were sorted into GFP+ and GFP- populations (Figure 1B) and enrichment for ciliated cells in the GFP+ population was demonstrated by immunofluorescence labeling of cilia (Figure 1C). RNA from GFP+ and GFP- samples was then hybridized to mouse predicted-exon oligo microarrays and compared against a universal mouse reference RNA pool. This yielded the transcriptional profile of GFP+ MTECs (ciliating or ciliated) at ALI+4 and ALI+12 and of non-ciliated, GFP- MTECs.

**Figure 2:**
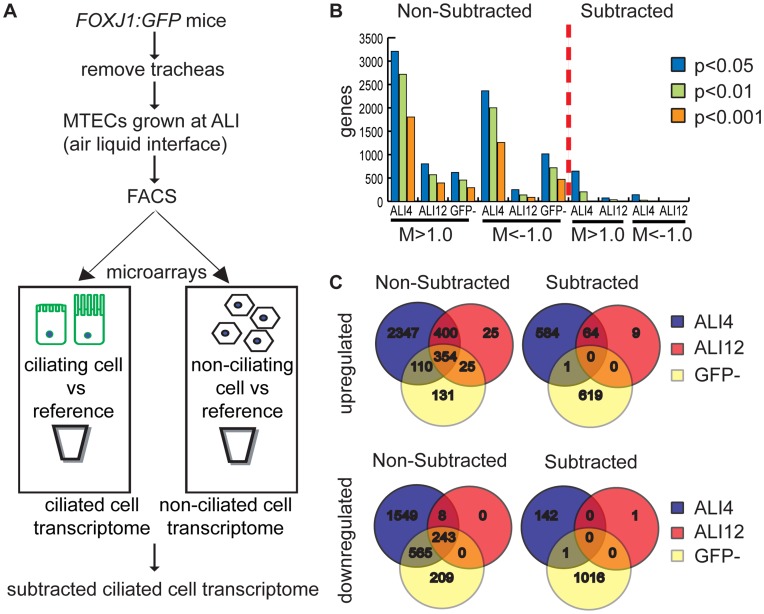
Subtractive analysis of MTEC microarray data. (A) Experimental flowchart detailing microarray analysis. (B) The number of differentially expressed genes in ciliated MTECs at adj. p-values <0.05, <0.01 and <0.001 before subtraction (non-subtracted) and after subtraction. The non-subtracted gene list is the transcriptional profile of GFP+ cells compared to a universal mouse reference RNA. The subtracted gene list was derived analytically by comparing the transcriptional profile of GFP+ cells to that of GFP- cells from MTEC cultures. The number of genes that show 2-fold or greater differential expression and have adjusted p-values of 0.05, 0.01 and 0.001 are shown before and after subtractive analysis. After subtraction, 649 genes were significantly upregulated at ALI+4 with respect to GFP- cells, and 73 genes at ALI+12. 143 genes were downregulated at ALI+4 and none at ALI+12 (adj. p<0.05). (C) The overlap of significantly upregulated and downregulated genes (adj. p<0.05) from ALI+4, ALI+12 and GFP- pools decreases after subtraction. The GFP- group in both panels represents genes that are differentially expressed in non-ciliated cells relative to the universal reference RNA. M = log_2_(fold change).

The experimental design is shown in Figure 2A. The GFP+ MTECs represent a unique cell type that would be expected to have several transcriptional signatures, including those common to lung epithelial cells in general. To identify the signature specifically associated with ciliogenesis, ALI+4 (Table S1) and ALI+12 (Table S2) transcriptomes were generated by subtracting the non-ciliated, GFP- cell transcriptome (Table S3) from the transcriptomes of GFP+ ciliated cells at both timepoints. This resulted in a set of 649 unique upregulated genes at ALI+4 and 73 unique upregulated genes at ALI+12, where significant differential expression was defined as a fold-change cutoff of 2-fold or greater in addition to an adjusted *P* value <0.05 (Figure 2B). Importantly, subtraction decreased both the number of differentially expressed genes at ALI+4 and ALI+12 (Figure 2B) as well as the overlap between genes that are differentially expressed in both ciliated and non-ciliated cells relative to the reference (Figure 2C).

### Functional Annotation of MTEC Transcriptome

We next assessed the effects of subtraction on the functional annotations recovered from these gene sets. Bioinformatics tools provided online at DAVID (Database for Annotation, Visualization and Integrated Discovery, http://david.abcc.ncifcrf.gov/) were used to mine known and predicted functional annotation information from databases including Gene Ontology (GO), SMART, INTERPRO and KEGG Pathway, for the subsets of genes expressed 5-fold or greater in *FOXJ1:GFP*- cells and *FOXJ1:GFP*+ cells at ALI+4 and ALI+12 (Table S4) [25]. As expected, *FOXJ1:GFP*- MTECs were enriched in genes associated with epithelial cell functions, including epithelial cell differentiation and cell adhesion, as well as cellular compartments common to all epithelial cells, such as the apical junction complex, desmosomes and tight junctions. The functional clusters most enriched in *FOXJ1:GFP*+ cells at ALI+12 were also associated with epithelial cell functions and structures. Additionally, the *FOXJ1:GFP*+ ALI+12 subset was enriched in components of the glutathione S-transferase pathway, which may be upregulated in these cells to aid in detoxification during the approximately 17–20 days of *in vitro* culture that the MTEC primary culture and differentiation protocol requires. However, after subtraction of the *FOXJ1:GFP*+ ALI+12 transcriptomic data, a strong enrichment in groups characteristic of cilia and microtubules was revealed, indicating that this analytical method successfully removes genes that are common to epithelial cells, leaving genes that are specific to ciliated cells. A similar strong enrichment in cilia and microtubule-related functional groups was observed in *FOXJ1:GFP*+ ALI+4 cells. The enrichment was apparent without subtraction, although subtraction increased the number of genes found in these clusters as well as the statistical significance associated with enrichment of these clusters. Based on these results, we focused our subsequent work on the subtracted dataset, and references to differentially expressed genes at ALI+4 and ALI+12 pertain to this subtracted data. The full dataset with subtracted and non-subtracted data can be accessed online at GEO (GEO Accession number GSE42500).

### Comparison to Related Datasets

Several approaches have been taken to identify the protein components of centrosomes and cilia. These include comparative genomics and proteomics approaches, transcriptional assays, and proteomics analyses of motile and non-motile cilia and basal bodies and centrosomes in a variety of ciliated organisms including Trypanosoma, Chlamydomonas, Caenorhabditis, Drosophila, Dictyostelium, mice and humans [28], [29], [30], [31], [32], [33], [34], [35], [36], [37], [38], [39], [40], [41], [42], [43], [44], [45], [46]. We assessed the quality of our dataset through comparison to these other published datasets.

First, we assessed the overlap of highly upregulated genes from our ciliated MTEC dataset with two existing cilia proteome datasets, which are compendia of ten proteomic, genomic and comparative genomic studies of cilia components [47], [48]. These include a transcriptomic analysis of Chlamydomonas during flagellar regeneration [29], a process similar to growth of motile cilia in MTEC cultures, as well as a proteomic analysis of ciliary axonemes isolated from human bronchial epithelial cells [36]. In the ALI+4 dataset, 127 of 288 genes that were upregulated 10-fold or more (44%) were previously identified in one or more studies in the cilia proteome databases, and in the ALI+12 dataset, 67/123 (54%) of such genes were previously identified (Table S5). Thus, almost half of the genes that were upregulated 10-fold or more in ciliated MTECs at ALI+4 and ALI+12 were not found in any of the previous studies included in the cilia proteome databases. Second, we tested whether our dataset was enriched for targets of the FOXJ1 transcription factor, as predicted by our use of *FOXJ1:GFP* to isolate ciliating cells. Among 86 putative FOXJ1 targets identified in zebrafish and Xenopus [49], [50], the mouse orthologs of 42 of these were identified as upregulated in the ALI+4 dataset, and 31 of these were upregulated in the ALI+12 dataset (Table S5). Lastly, we interrogated our dataset using a test set of 99 mouse genes predicted to be linked to cilium formation by their expression in tissues that contain numerous ciliated cells such as olfactory epithelium, testes, vomeronasal organ, trachea, and lung [46]. 39 out of the 99 genes identified by tissue expression patterns were significantly upregulated (p<0.05) at ALI+4, and 16 out of 99 genes were significantly upregulated at ALI+12 (Table S5). Interestingly, when we performed the reverse analysis, we found that only 6% of upregulated genes from ALI+4 were previously identified in this study, whereas 21% of upregulated genes from ALI+12 were previously identified. This likely reflects the fully differentiated state of the majority of ciliated cells found in the tissues assayed in previous studies, and suggests that our dataset may hold clues to the early stages of ciliogenesis that have not been revealed by other studies.

### *In Situ* Localization of Upregulated Proteins in the Human Protein Atlas

We next assessed which of the proteins encoded by genes that were upregulated in our ciliated MTEC datasets can be detected at the cilia or the basal bodies of multiciliated cells. To do so, we scanned immunohistochemistry data from the Human Protein Atlas (HPA), an online compendium of tissue microarray data from normal tissues, pathology specimens and cell lines [51]. The HPA dataset consists of tissue sections stained with antibodies raised against human proteins. HPA data were recently used to validate results from a mass spectrometry-based proteomics screen for candidate centrosome proteins [43], as well as a second *in silico* identification of cilia proteins based on tissue RNA expression [52]. We used this tool to evaluate MTEC candidate genes.

The HPA includes tissue sections from bronchus, nasopharynx and the fallopian tube, which are tissues containing multiciliated epithelia. We found that we could discern four patterns of antibody staining in these sections relevant to the structure and function of ciliated epithelial cells: 1) cilia, visible as a tuft on the apical surface; 2) basal body layer, visible as a line at the junction of the ciliary tuft and the cell body, 3) the nucleus of ciliated cells, 4) whole cell staining of ciliated cells, relative to adjacent non-ciliated cells. We analyzed 8,038 HPA images representing 923 proteins whose orthologous mouse proteins are encoded by genes that were upregulated 4-fold or more in ciliated MTEC (Table S6). Of the 923 proteins represented, 504 (55%) revealed staining of ciliated cells: 195 (21%) localized to basal bodies, 163 (17%) localized to cilia, 22 (2%) localized to ciliated cell nuclei and 143 (15%) localized to other regions of ciliated cells in at least one of the three tissues examined. These data confirm by an independent method that a high percentage of genes detected as differentially expressed in ciliated MTECs by our subtractive transcriptional analysis localize to structures specifically in the ciliated epithelial cells of multiple human tissues.

### Functional and Physical Association of Proteins Upregulated during Ciliogenesis

**Figure 3:**
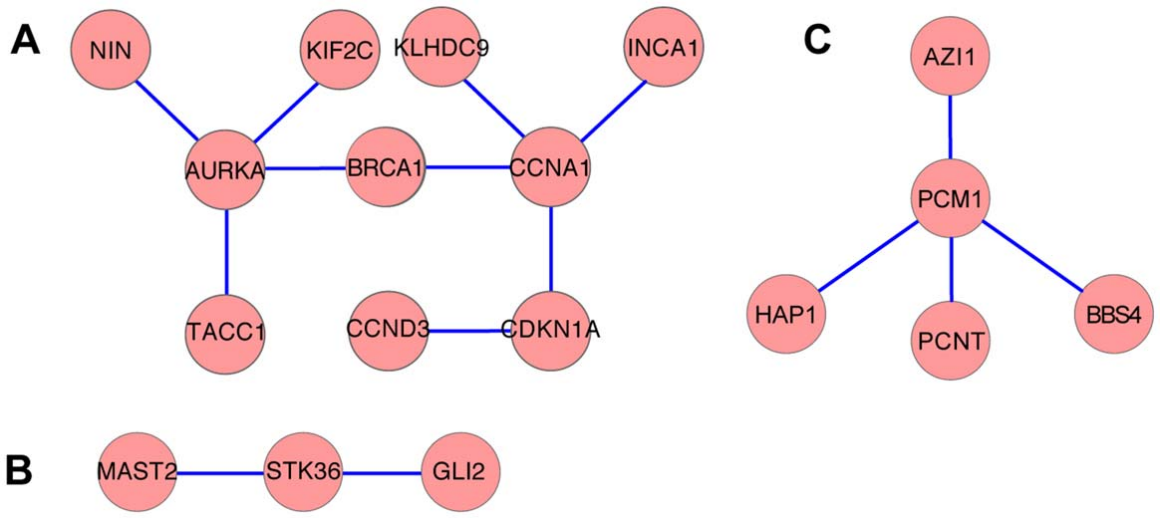
Functional mapping of candidate centrosome/cilia proteins and identification of novel centrosome components. Candidate genes were organized by known physical and genetic interactions mined from Mitocheck, the Human Protein Reference Database and BioGrid. Clusters containing genes associated with cell cycle control (A), hedgehog signaling (B) and the PCM1 complex (C) are shown.

Other tools we used to evaluate the ciliated MTEC transcriptomic data were Mitocheck (www.Mitocheck.org), the Human Protein Reference Database (HPRD) [53] and the biological general repository for interaction datasets (BioGrid) repository [54]. Previously uncharacterized proteins that were upregulated during ciliogenesis were organized by known physical and genetic interactions (File S1). Mapping in this manner revealed nodes of genes with defined functional or structural links to cilia or centrosomes, such as cell cycle regulators (*Aurka*, *Brca1*, *Ccna1,* Figure 3A), *hedgehog* signaling components (*Stk36*, *Gli2*, Figure 3B), and the PCM1 complex (*Pcm1, Bbs4, Hap1*, Figure 3C) [55], [56], [57], [58], [59], [60]. Based on these results, we identified the uncharacterized gene *Klhdc9* to be a potential candidate for the cell cycle control of ciliogenesis, and *Mast2* to participate in hedgehog signaling. Additionally, the cluster containing the PCM1 complex contains AZI1, also known as CEP131 in humans and Dilatory (DILA) in flies, which are proteins that are involved in ciliogenesis [61]–[62]. AZI1, like PCM1 and BBS4 [63], [64], has recently been confirmed to be a component of centriolar satellites [65]. This example illustrates how combining the transcriptional data on MTEC ciliogenesis with publicly available and curated protein interaction data can help to identify *bona fide* components of structural and functional complexes.

### A Common Set of Genes Associated with Centriole Duplication and Basal Body Generation

**Figure 4:**
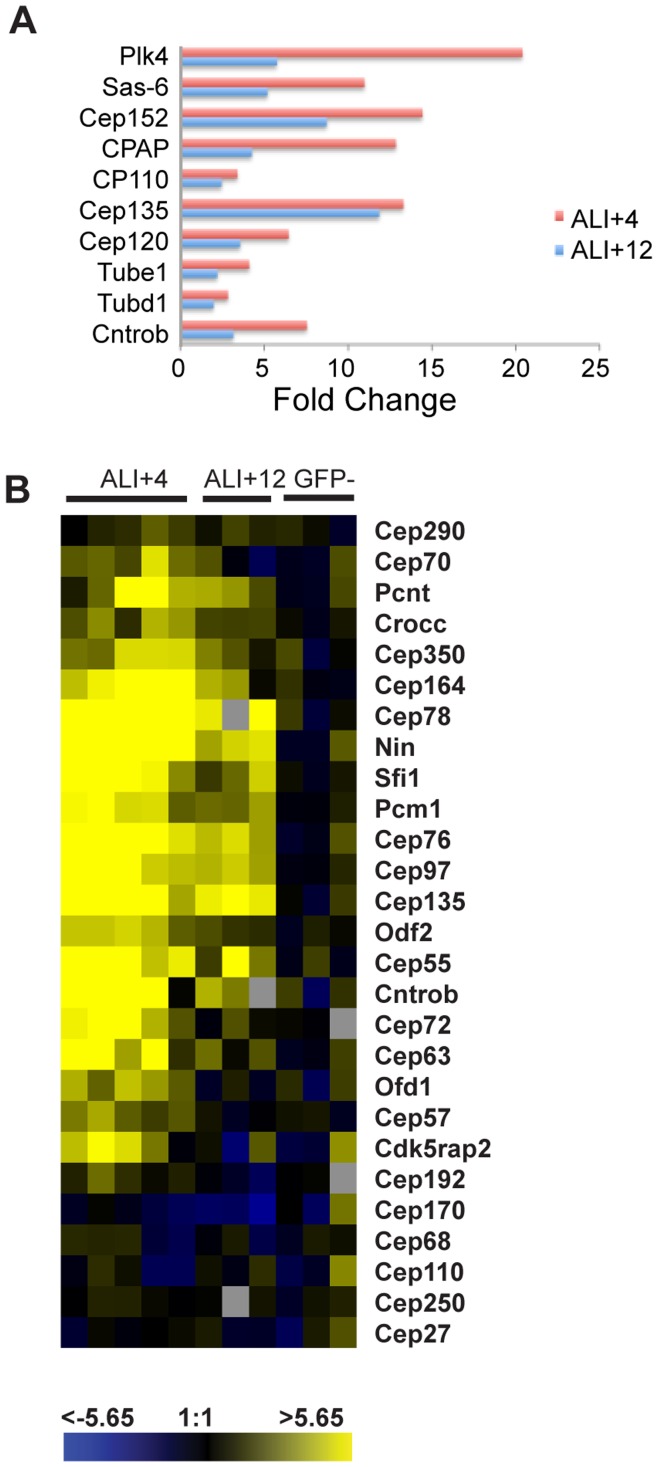
Genes encoding centrosome proteins are differentially regulated during basal body formation. (A) Upregulation of regulatory and structural proteins required for centriole duplication in cycling cells and (B) heat map of putative and known centrosomal components. Genes are shown in rows and replicate arrays are shown in columns. Three biological replicates were performed for ALI+4, ALI+12 and GFP-. In addition, two technical replicates were performed for two of the ALI+4 biological replicates (columns 1,2 and 4,5) for a total of five columns. Data were zero-transformed against non-ciliated (GFP-) cells. The scale indicates the fold change in expression (log_2_). Grey boxes represent gene spots that failed to pass quality control filters for the indicated array.

The basal bodies of ciliated epithelial cells are morphologically similar to the centrioles in cycling cells, but the degree of that similarity with respect to composition and mechanism of formation is unclear. To explore this relationship, we first asked whether the genes encoding a core set of proteins known to be required for centriole duplication in cycling cells are upregulated during ciliogenesis. This set includes PLK4, SAS-6, CENPJ/SAS-4, CEP152, CEP120, CEP135, CP110, centrobin, delta-tubulin and epsilon-tubulin, all of which are physically associated with centrioles [18], [66], [67], [68]. All of the genes encoding these proteins were upregulated during MTEC ciliogenesis (Figure 4A), suggesting that centriole formation in cycling cells and basal body duplication in ciliated epithelial cells share a common mechanism. As predicted, the degree of the upregulation for core centriolegenesis genes was greater at ALI+4 when the majority of ciliating cells are generating basal bodies than at ALI+12, when centriolegenesis is complete in most cells [69]. Most striking among these results was that *Plk4*, a master regulator of centriole formation ([12], [70], [71]), was upregulated more than 20-fold at the ALI+4 timepoint (Figure 3A).

In addition to genes involved in centriole duplication, many other genes encoding known centrosomal proteins were also upregulated in the ciliated MTEC dataset (Figure 4B). In some cases the upregulated proteins had only previously been reported to play a role in centrosome functions in cycling cells seemingly unrelated to basal body function. For example, CEP63 and CEP72 have been shown to function in formation of bipolar spindles in mitotic cells [72] ^,^ [73]. We speculate that the upregulation of such proteins during ciliogenesis in ciliated MTEC reflects a cilium-related function in the interphase centrosome of cycling cells, in which the mother centriole acts as a basal body for the primary cilium. Some centrosomal components identified in cycling cells were not induced during MTEC ciliogenesis. These include CEP170, CEP192, CEP250/C-NAP1, CEP27/HAUS2 and CEP68, all of which are thought to have functions associated with centrosomes, and not basal bodies, including microtubule and spindle organization, recruitment of pericentriolar material and centrosome cohesion [74], [75], [76], [77], [78], [79]. Similarly, the gamma-tubulin ring complex mediates microtubule nucleation at the centrosome [80], [81], and most of the members of this complex were not significantly upregulated relative to non-ciliated cells, consistent with the notion that a limited subset of centrosome functions is associated with the centrioles serving as basal bodies in ciliated MTECs.

**Figure 5:**
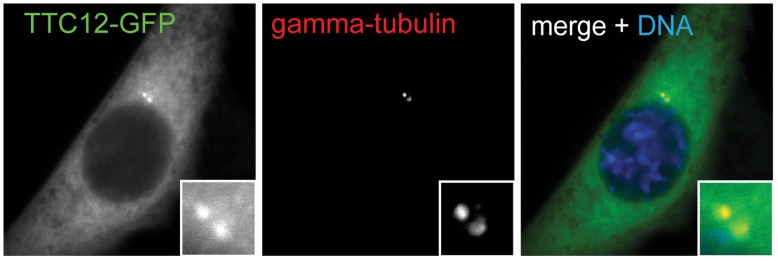
Identification of a novel centrosome component. NIH/3T3 cells were transfected with a plasmid encoding TTC12-GFP. TTC12-GFP localizes to the centrosome as shown by overlap with gamma-tubulin staining. Green, TTC12-GFP. Red, gamma-tubulin.

In the interest of identifying new components of the centrosome in our dataset, we reasoned that such genes might have the pattern of high upregulation at ALI+4, during basal body formation, and lower expression at ALI+12, after ciliogenesis is complete. TTC12 (tetratricopeptide repeat domain 12) is one such protein that fits this criterion and was previously identified by comparative genomics of ciliated and non-ciliated organisms [30]. *Ttc12* was upregulated 2.3-fold at ALI+4 and decreased to 1.5-fold at ALI+12 (Tables S1, S2). To test whether TTC12 is centrosome protein, TTC12-GFP was expressed in NIH/3T3 cells. We found that TTC12 localizes to the centrosome as defined by gamma-tubulin labeling (Figure 5), demonstrating that the transcriptional profile of ciliated MTEC can be used to identify novel centrosome components.

### Identification of Novel Links to Human Disease

**Figure 6:**
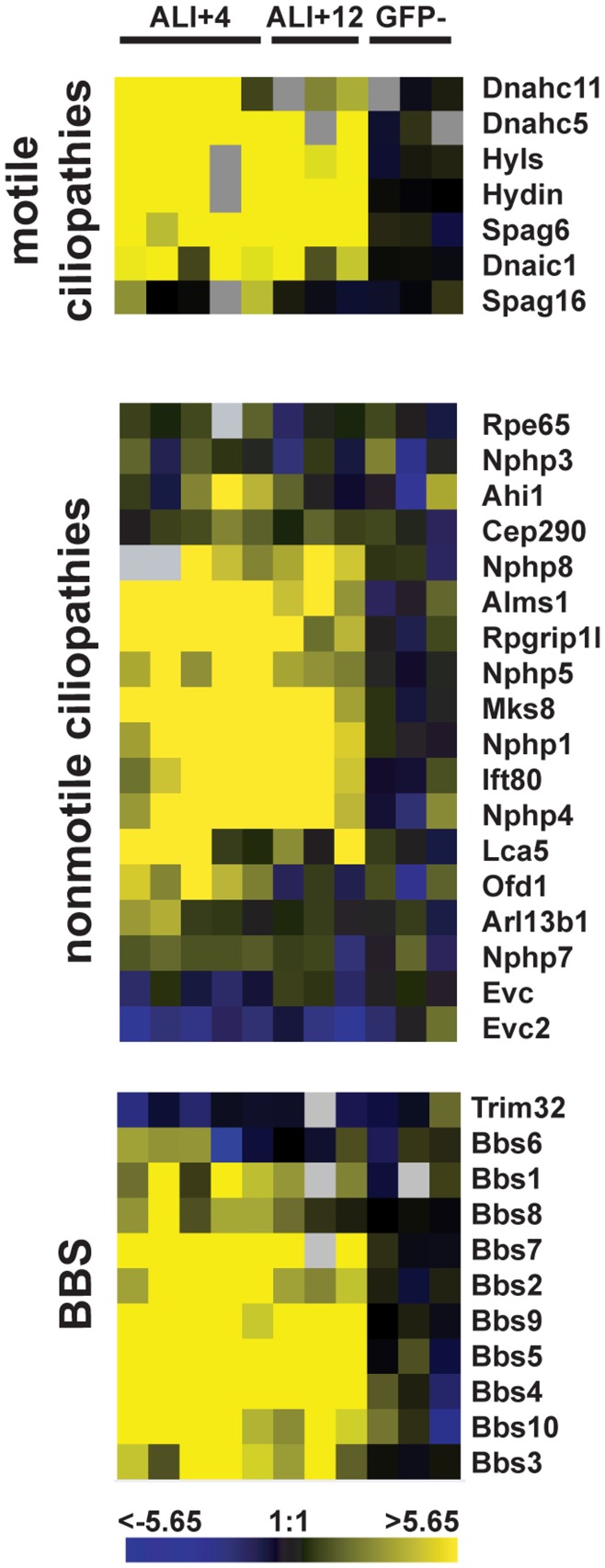
Differential regulation of disease genes associated with motile and nonmotile ciliopathies. Heat maps showing the expression of genes associated with motile (top) and nonmotile (middle) ciliopathies, as well as genes linked to Bardet-Biedl syndrome (bottom), a model ciliopathy.

Historically, the human diseases associated with cilia have been considered as two separate groups: those associated with defects in the beating of motile cilia, and those associated with non-motile cilia, i.e. the primary cilium. It is unclear whether the proteins associated with the signaling functions of non-motile cilia are unique to those cilia; however, the motile flagella of Chlamydomonas and its components clearly have signaling functions [82], [83], [84], and motile cilia in mammalian ciliated epithelia are competent for specific signaling events [85], [86]. We examined the transcriptional data for genes with known links to diseases of motile and non-motile cilia. As expected from the source of cells in our experiments, many of the genes associated with motile cilium diseases were upregulated in the ciliated MTEC dataset (Figure 6 top panel). Notably, many genes associated with non-motile cilium diseases were also upregulated, including *Rpgrip1l/Nphp8, Alms1, Nphp1* and *Nphp4, Mks8, Ofd1* and *Lca5* (Figure 6 middle panel). This suggests that these genes encode components common to nonmotile and motile cilia, and that mutations in them might also display a phenotype in motile cilia. This is consistent with the results for the Bardet-Biedl syndrome (BBS) genes, most of which are similarly upregulated in the ciliated MTEC dataset, and have been linked to defects in both motile and non-motile cilia [7] (Figure 6 bottom panel).

We examined three cases in which a gene upregulated in ciliated MTEC had been previously associated with a mammalian disease phenotype, but not yet associated with motile cilia, primary cilia, or the centrosome.

#### Mdm1

**Figure 7:**
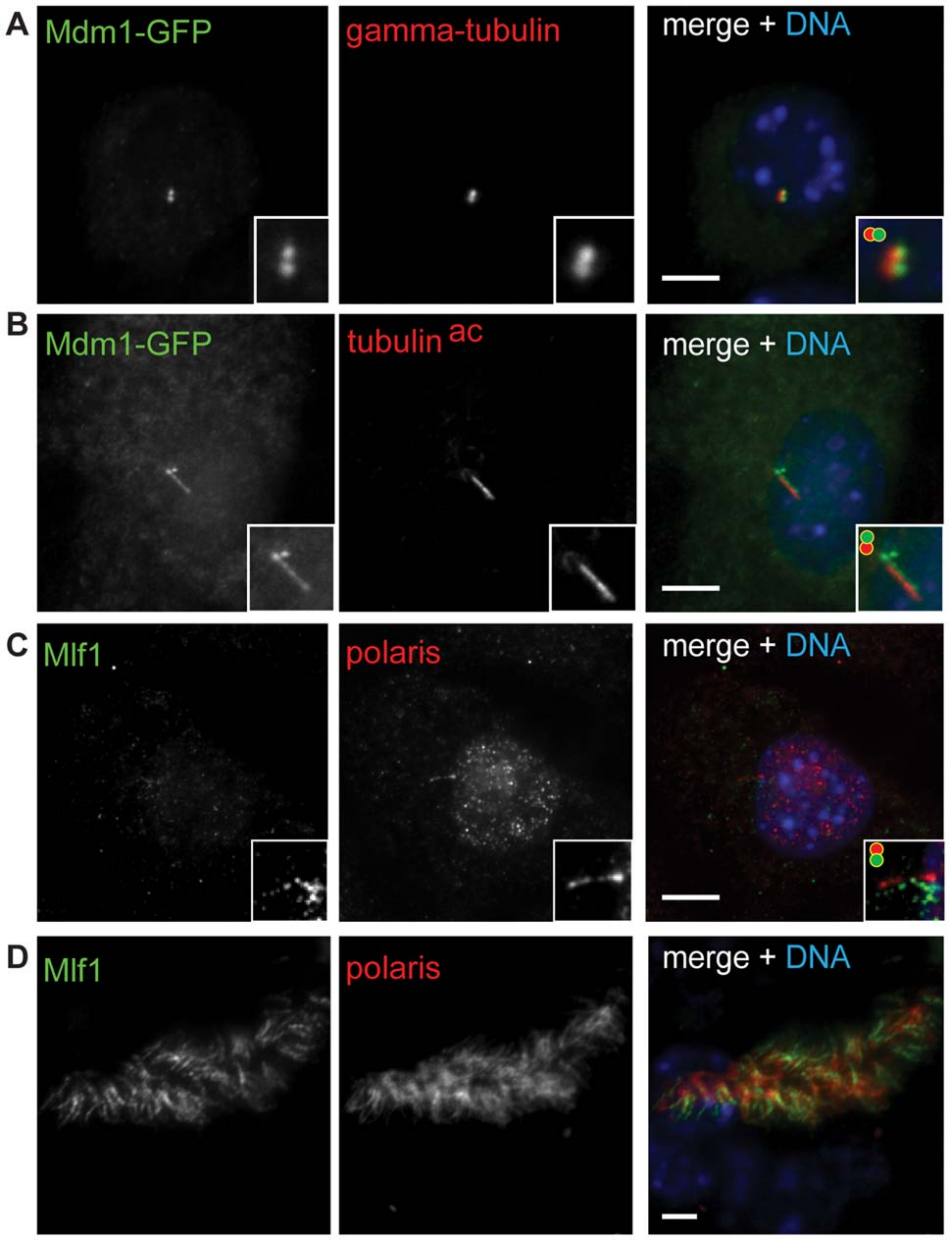
Identification of new links between centrosomes, cilia and human disease. (A, B) NIH/3T3 cells were transfected with a plasmid encoding MDM1-GFP. MDM1-GFP localizes to centrosomes (A) and the primary cilium (B). NIH/3T3 cells and MTECs were stained for MLF1 with an anti-MLF1 antibody. MLF1 localizes to the primary cilium in NIH/3T3 cells (C) and motile cilia in MTECs (D). Insets show higher magnification of merged images, slightly offset as indicated by circles in upper left. Scale bars, 5 µm. Green, GFP (A, B); MLF1 (C, D). Red, gamma-tubulin (A); acetylated tubulin (B); polaris (C, D).

The connecting cilium of the photoreceptor outer segment is a specialized type of cilium that shares many features and components with motile cilia and primary cilia [87], and many genes liked to retinopathy encode proteins that localize to the connecting cilium [88]. A positional cloning approach identified a nonsense mutation in *Mdm1* in the *arrd2* mouse model for age-related retinal degeneration [89], indicating the *Mdm1* gene product may be important for retinal function. *Mdm1*, which is >60-fold upregulated in ciliated MTECs (Tables S1, S2), has also been identified in proteomic analysis of human centrosomes [38] and mouse photoreceptor sensory cilia [90] and is conserved in organisms with ciliated epithelial cells, suggesting that it is a component of cilia and or basal bodies. To test whether MDM1 is a centrosome/cilia component we expressed a MDM1-GFP fusion protein in NIH/3T3 cells. MDM1-GFP localized to centrosomes, as determined by colocalization with gamma-tubulin (Figure 7A) and to the primary cilium, as determined by colocalization with acetylated alpha-tubulin (Figure 7B). Based on these results we suggest that the age-related retinal degeneration phenotype observed in the *aard2* mouse results from a defect in connecting cilium function.

#### Mlf1

*Mlf1* (*myeloid leukemia factor 1*) is associated with myeloid leukemia; *Mlf1* is fused to nucleophosmin (*Npm1*) in 1% of acute myeloid leukemia (AML) cases [91] and is overexpressed in 25% of cases of AML associated with myelodysplastic syndrome [92]. Based on observed interactions with p53, it has been suggested that defects or dysregulation of *Mlf1* might perturb cell cycle regulation and contribute to malignant transformation [93]. We identified *Mlf1* as being >30-fold upregulated in ciliated MTECs (Tables S1, S2). In addition, MLF1 has been identified in proteomic analyses of the centrosome and connecting photoreceptor cilium [36], [90]. To test the localization of MLF1, we stained NIH/3T3 cells for MLF1 using a commercially available antibody (H00004291-B01, Abnova). Faint MLF1 staining was observed in primary cilia, as determined by colocalization with the intraflagellar transport component polaris (Figure 7C). MLF1 also labeled the length of motile cilia in MTECs (Figure 7D) and in some cells appeared to be concentrated at the apical tips (not shown). These results indicate that MLF1, a putative regulator of p53 and the cell cycle, is a component of both primary and motile cilia. Interestingly, the closely related protein MLF2 was identified in ciliary proteomic studies [36], [90], and tissue labeling in the HPA indicates that MLF2 localizes to cilia in ciliated epithelial cells (Table S6). We also note also that the putative Drosophila ortholog of MLF1/2 interacts with Su(fu), a component of the hedgehog signaling pathway [94] that localizes to the primary cilium in mammals [59].

#### Dyx1c1

**Figure 8:**
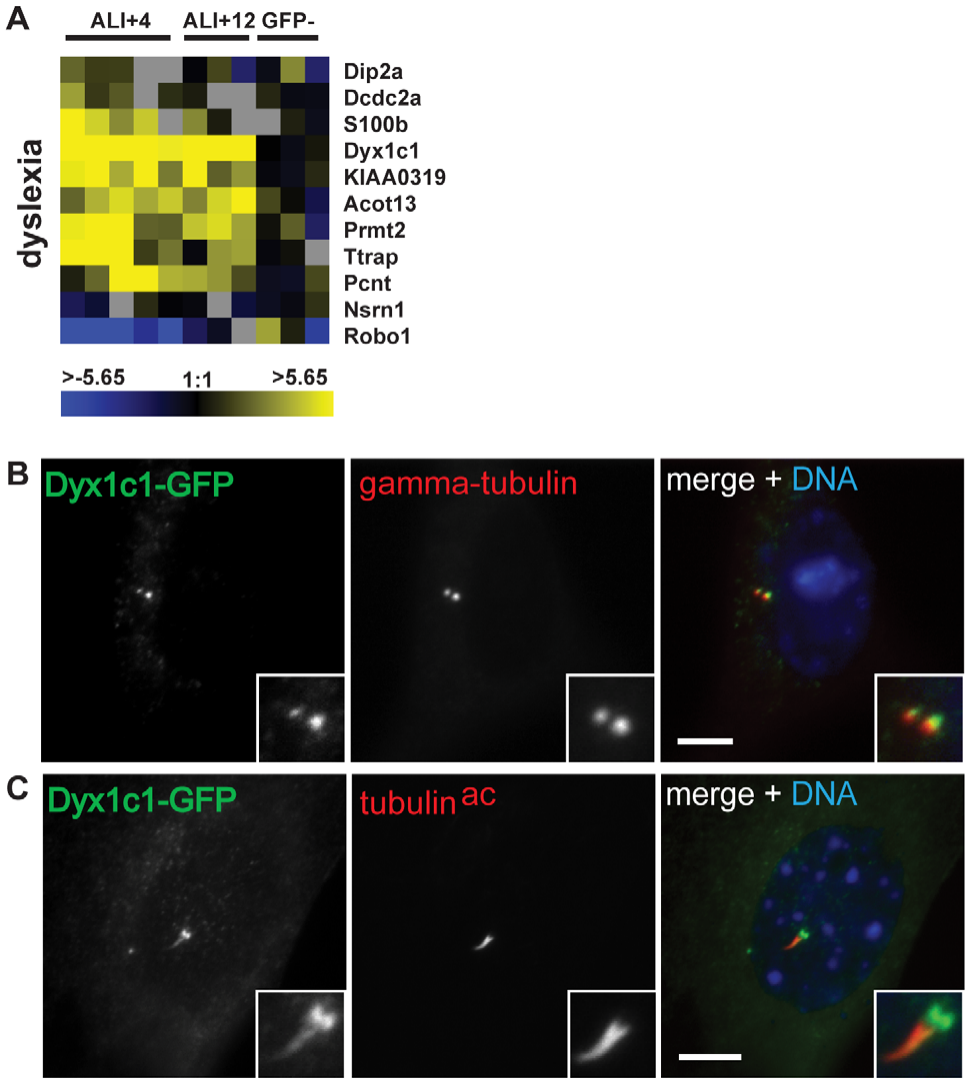
A dyslexia candidate disease gene is transcriptionally upregulated and localizes to the centrosome. (A) Heat maps depicting the expression of dyslexia candidate genes in ciliated MTECs. *Dyx1c1* is among the most highly upregulated genes, and *Robo1* is strongly downregulated. (B-D) A DYX1C1-GFP fusion protein was expressed in NIH/3T3 cells. DYX1C1-GFP localizes to the centrosome (B) and in some cells the primary cilium (C). Insets show positively staining regions at higher magnification. Scale bars, 5 um. Green, GFP (B, C). Red, gamma-tubulin (B); acetylated tubulin (C).

Neurocognitive impairments have been observed in individuals with ciliopathies, but the mechanistic links between the neurodevelopmental and neurocognitive diseases and cilium function are poorly understood [88]. Dyslexia is one such neurocognitive impairment that has recently been linked to primary cilia through the localization of the product of the candidate dyslexia susceptibility gene *Dcdc2* to primary cilia in primary rat neurons, and the effect of its overexpresion on cilia length [95]. Several genetic loci in addition to *DCDC2* have been linked to dyslexia susceptibility [96]. We noted that nine of ten candidate dyslexia genes present in three of the dyslexia-associated genomic regions [97], [98], [99] were differentially regulated in ciliated MTECs (Figure 8A). One of these genes, *Dyx1c1* (Dyslexia Susceptibility Candidate 1), is linked both genetically and mechanistically to dyslexia [99], [100]. *Dyx1c1* was upregulated more than 16-fold at ALI+4 in ciliated MTECs. It also was previously identified in a comparative genomics screen for genes that are found only in organisms having cilia [30], in an *in silico* study that used public microarray data to identify potential cilia components [45], and identified as interacting with the cytoskeleton in an assessment of molecular networks [101]. Based on these results, we examined the localization of GFP-tagged versions of rat DYX1C1 [102] that we expressed in NIH/3T3 cells. DYX1C1-GFP colocalized with gamma-tubulin at the centrosome (Figure 8B). Although the centrosome localization was most prominent, DYX1C1-GFP also localized to the primary cilium in some cells (Figure 8C). These results suggest that the neuronal migration defect of DYX1C1 depletion may reflect a function of this protein in the centrosome or cilium, adding to the growing list of such proteins that have been linked to diseases of the brain [103].

## Discussion

We determined the transcriptional profile of ciliated tracheal epithelial cells undergoing differentiation *in vitro*. Previous studies focused on ciliated cells or single-cell organisms, including analyses of human bronchial epithelia [44], brain ependyma [42] and of Chlamydomonas flagellar regeneration [29] have yielded useful information, however, the combination of the unique properties of the ciliated MTECs with the cell-type specificity achieved by cell sorting make this a particularly powerful dataset. The upregulated genes comprise a dataset that both reveals aspects of function in this unique ciliated cell type, and identifies new proteins of the centriole, centrosome and cilium that include disease-associated proteins that were not previously known to be associated with centrosome and cilium defects. Here we consider the implications of these results for centrosome and cilium biology and human disease.

Ciliated epithelial cells produce hundreds of centrioles and motile cilia during the course of differentiation, whereas cycling cells produce only two centrioles and one non-motile primary cilium per cell cycle. Centriole formation in ciliated epithelial cells has been shown by electron microscopy to be morphologically distinct from that in cycling cells, with many centrioles forming simultaneously in close proximity to deuterosomes, structures unique to differentiating ciliated epithelial cells [19]. Although several previous studies have shown that particular centriole and centrosome components are upregulated during ciliated epithelial cell differentiation, the relationship of the critical regulators of centriole formation in cycling cells to the amplification process in ciliated epithelial cells was not clear. Our results show that the core set of proteins required for centriole duplication in cycling cells is upregulated during basal body formation in ciliated MTECs. Most importantly, this includes Cyclin A (*Ccna1*), which is required for centriole formation [104], PLK4 and SAS-6, two proteins known to be capable of inducing the formation of multiple centrioles when overexpressed [71], [105], and CEP152/ASL, which is required for normal centriole duplication and PLK4-induced overduplication in cultured human and fly cells [106], [107]. Consistent with the notion that upregulation of genes reflects a role in the unique events of ciliated MTEC differentiation, we have previously shown that SAS-6 is required for centriole formation in ciliated MTECs [17]. We suggest that basal body formation in multiciliated cells likely occurs by the same mechanism as in cycling cells, but that the tight control over centriole number in cycling cells is bypassed by strong upregulation of PLK4, allowing the formation of many centrioles at once. However, it remains possible that some unique aspects of basal body formation, such as the presence of deuterosomes, might involve gene products specifically expressed in multiciliated cells.

We found that expression of many genes associated with centriole formation was highest at ALI+4 when centrioles, or basal bodies, were forming and declined at ALI+12 when centriole formation was largely completed. We used this as a screening criterion for the identification of new centrosome components amongst the previously uncharacterized genes in the dataset. In this first-pass analysis, TTC12 was identified to be a new centrosome component. In addition, we used data from the Human Protein Atlas to assess the localization of proteins from 923 upregulated genes, and found that approximately 45% localized specifically to multiciliated epithelial cells in the tissues examined, and approximately 38% localized to the basal body layer, to cilia, or to both. Since many of these proteins were previously uncharacterized these results greatly expand the number of potential components of the centrioles and cilium. Although some of these will be specific to motile cilia or basal bodies in multiciliated cells, it is likely that many are general components of centrioles and cilia, based on the high representation of known centriolar and ciliary proteins in the dataset, and on the high frequency with which genes identified here have been identified in at least one of the other proteomic and genomic datasets associated with the centrosome and/or primary cilium. We also drew on protein interaction information from publicly available databases to map out known interactions between proteins that are upregulated during ciliogenesis, most of which have unknown function. The value of such an analysis was demonstrated by correctly identifying an interaction between centriolar satellite protein AZI1/CEP131 and the PCM1 complex, all of which are highly expressed during differentiation of multiciliated cells.

Among the genes upregulated during ciliogenesis in ciliated MTEC, we have identified several associated with human disorders. The disorders include those known to be associated with centrosome/cilium dysfunction, such as retinal degeneration, but also others not previously associated, such as leukemia and dyslexia. Neurocognitive deficits in general are observed in patients with mutations affecting centrosome or cilium function, ranging in severity from microcephaly and lissencephaly, in which the brain or segments of it are greatly reduced in size or complexity, to more subtle neurological impairments such as schizophrenia and bipolar disorder [108] ^,^ [109]. In this context it would not perhaps be surprising that a reading disability would have a centrosome/cilium defect as its root cause. We show here that DYX1C1 is physically associated with the centrosome and the cilium, and also note that another strong dyslexia candidate gene from human genetic studies, *DCDC2*, encodes a protein that is a component of the primary cilium that can impact cilia length when overexpressed [95]. The molecular basis for dyslexia is unknown, but these candidates have both been shown to be required for neuronal migration in the cortex, providing one possible mechanism for development of the disease [110].

The set of human diseases referred to as ciliopathies has received much attention recently, and their analysis is an outstanding example of the reciprocal interaction between clinical and basic research. We note that the ciliated MTEC transcriptome dataset described here might be particularly useful for extending the correlations between human phenotype and centrosome/cilium function because it is based on a mammalian gene set, and is focused on a cell type that has an extreme dependence on the centrosome/cilium for its physiological function. Here we have established connections between genes identified by human or other mammalian genetic studies, upregulation of the genes in this specialized tissue, and localization of the cognate proteins to the centrosome/cilium. Future work will determine whether the pathologies caused by mutation of these genes result from defects in centrosome/cilium function, or alternatively whether localization of the disease-associated protein to the centrosome/cilium is important for the function of that particular protein or pathway.

## Supporting Information

Table S1 **Subtracted ALI+4 transcriptome.**
(XLSX)

Table S2 **Subtracted ALI+12 transcriptome.**
(XLSX)

Table S3 **GFP- transcriptome.**
(XLSX)

Table S4 **Top 5 functional annotation clusters in** ***FOXJ1:GFP*** **+ (ALI+4 and ALI+12) and** ***FOXJ1:GFP*** **- MTECs.**
(XLSX)

Table S5 **Overlap of the ciliated MTEC transcriptome with other proteomic, transcriptomic and genomic studies of the cilium and centrosome.**
(XLSX)

Table S6 **Mining the Human Protein Atlas for localization of human orthologs of candidate ciliated MTEC proteins in multiciliated human tissues.**
(XLSX)

File S1 **Inferring function of candidate ciliogenesis proteins by mapping known physical and genetic interactions.**
(CYS)
